# Increased Flexibility in Lab-on-Chip Design with a Polymer Patchwork Approach

**DOI:** 10.3390/nano9121678

**Published:** 2019-11-25

**Authors:** Denise Pezzuoli, Elena Angeli, Diego Repetto, Patrizia Guida, Giuseppe Firpo, Luca Repetto

**Affiliations:** Department of Physics, Università degli Studi di Genova, Via Dodecaneso 33, 16146 Genova, Italy

**Keywords:** nanofluidic device, polydimethylsiloxane, polymeric device, nanochannel, nanofabrication

## Abstract

Nanofluidic structures are often the key element of many lab-on-chips for biomedical and environmental applications. The demand for these devices to be able to perform increasingly complex tasks triggers a request for increasing the performance of the fabrication methods. Soft lithography and poly(dimethylsiloxane) (PDMS) have since long been the basic ingredients for producing low-cost, biocompatible and flexible devices, replicating nanostructured masters. However, when the desired functionalities require the fabrication of shallow channels, the “roof collapse” phenomenon, that can occur when sealing the replica, can impair the device functionalities. In this study, we demonstrate that a “focused drop-casting” of h-PDMS (hard PDMS) on nanostructured regions, provides the necessary stiffness to avoid roof collapse, without increasing the probability of deep cracks formation, a drawback that shows up in the peel-off step, when h-PDMS is used all over the device area. With this new approach, we efficiently fabricate working devices with reproducible sub-100 nm structures. We verify the absence of roof collapse and deep cracks by optical microscopy and, in order to assess the advantages that are introduced by the proposed technique, the acquired images are compared with those of cracked devices, whose top layer, of h-PDMS, and with those of collapsed devices, made of standard PDMS. The geometry of the critical regions is studied by atomic force microscopy of their resin casts. The electrical resistance of the nanochannels is measured and shown to be compatible with the estimates that can be obtained from the geometry. The simplicity of the method and its reliability make it suitable for increasing the fabrication yield and reducing the costs of nanofluidic polymeric lab-on-chips.

## 1. Introduction

Biomedical and environmental applications of fluidic chips for the detection of nanoplastics [1,2], proteins [3,4,5], viruses [6,7,8], bacteria [9,10] and DNA [11,12,13] rely on micro and nanofeatures that are the core elements of these devices for their capability to allow in-situ and real-time analysis of nanosize objects.

In order to achieve the desired functionality, these devices must be fabricated with nanometer-scale structures, i.e., dimensions close to the objects of interest. In particular, devices equipped with nanochannels that have, at least, one dimension under 100 nm allow exploiting several nanoscale transport phenomena that are crucial in single-molecule sensing experiments [14].

A paradigmatic example is DNA sensing: here biological nanopores such as α-haemolysin and Phi29 connector channel represent a good choice for their suitable dimension and bio-compatibility, but they are extremely fragile and susceptible to the environmental conditions [15,16]. For this reason, the integration of these pores in complex devices is difficult and not durable. Solid-state nanopores and nanochannels offer an attractive alternative that is characterized by high design versatility and operative soundness. Among the advantages of solid-state nanopores, one should mention the combination of well-defined geometry and size, the mechanical, thermal and chemical stability, and, usually, high suitability to optical and electrical experimental techniques [17,18,19]. Usually, solid-state nanochannels are produced on Si, SiO_2_ or glass substrates by using high-resolution nanopatterning techniques such as focused ion beam (FIB) milling, e-beam lithography (EBL) or laser machining [20,21,22,23,24,25]. These techniques provide excellent control of the geometry and high reproducibility but are usually expensive and not easily scalable to a production stage.

To overcome these limitations, we proved that the coupling of the previously mentioned nanopatterning techniques with polymer-based soft lithography methods [26,27,28] is an effective strategy for reducing the fabrication costs, yet maintaining good resolution for the nanofluidic structures. As an example, by using FIB milling for creating nanostructures on a micromachined silicon chip, and by replicating this master through soft lithography, we were able to fabricate hundreds of replicas of the same silicon master.

The main drawbacks related to the use of standard poly(dimethylsiloxane) (PDMS) for fabricating nanostructures are: (i) the high viscosity of the material and (ii) the “roof collapse” phenomenon. High viscosity, typically, limits the ability to reproduce the structures hindering a good replica fidelity [29]. “Roof collapse”, which occurs during the sealing process, can result in a partial or complete closure of the nanostructures. In fact, during this process, shallow nanochannels characterized by a low aspect ratio (height to width) tend to buckle on the closing substrate under the action of adhesion forces [30,31]. This mechanism can cause a reduction of the nanochannel dimensions, that, in the worst cases, results in a complete collapse of the structure, making the device unusable. In any case, a major alteration in the geometry strongly impacts the fabrication process by requiring accurate modeling to predict how the geometry will change. In extreme situations, when random effects sum up, all of this undermines the reproducibility and the production rate.

An obvious solution for preventing the risk of “roof collapse” would consist in increasing the Young’s modulus of the polymer used for the replica molding procedure. For example, hard poly(dimethylsiloxane) (h-PDMS), a material proposed in 2000 by Schmid et al. [32], has all the advantageous properties of PDMS, such as biocompatibility, optical transparency, and low cost, but it is characterized by a higher Young’s modulus of nearly 8.2 MPa compared to 1.8 MPa of standard PDMS [33]. So, if h-PDMS, is an effective material for replicating sub-100 nm structures [34], a major drawback of its increased stiffness, consists in the fact that cracks are often present on the polymeric replica surface [35], hindering the fabrication of large devices. These cracks are most probably produced when the replicas are peeled-off of the master and the applied momenta more easily exceed the ultimate shear stress of the device.

In this paper, we present a strategy called “focused drop-casting” for depositing h-PDMS, in order to make nanochannels more resistant to the collapse, without increasing the overall stiffness of the device. With this approach, we fabricate devices a few cm^2^ large with functional nanostructures. Specifically, we create an inhomogeneous material with the properties of h-PDMS in the critical regions (i.e., the regions with nanostructures), while maintaining the flexibility of standard PDMS out of them. This polymer patchwork approach allows obtaining stiff nanostructures and excellent replica flexibility at the same time.

## 2. Materials and Methods

### 2.1. Fabrication with the Patchwork Approach

**Figure 1:**
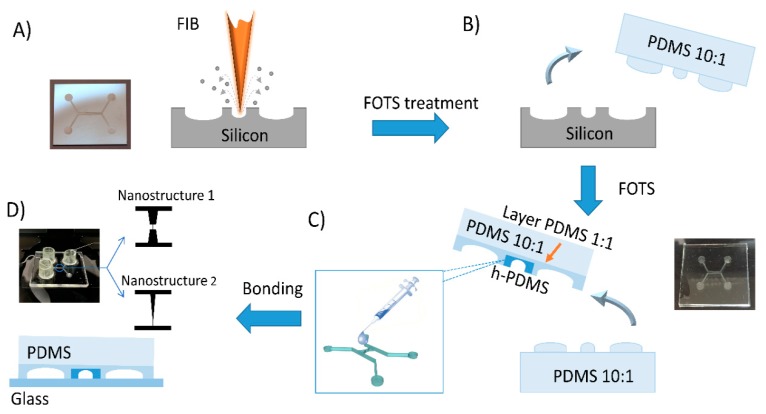
Schematic representation of the fabrication process of a polymeric nanofluidic device. (**A**) Silicon master patterned by focused ion beam (FIB) milling and treated, by vapor phase, with an antistiction layer of FOTS (1H,1H,2H,2H-perfluorooctyltrichlorosilane). (**B**) Negative replica fabrication followed by FOTS deposition. (**C**) Positive replica fabrication using PDMS 10:1, 1:1 and h-PDMS poured with “focused drop-casting” method, i.e., only one drop confined on the nanostructured region. (**D**) Bonding procedure and picture of the final nanofluidic device. The insets show a scheme of the geometries of the two nanostructures used for this study. Number 1 has an hourglass-shaped geometry, while number 2 has a funnel-shaped geometry.

In Figure 1 we show a schematic representation of the fabrication process that was used in this work. It is divided into four main steps: in the first one (Figure 1A) a FIB (CrossBeam 1540 xb, Carl Zeiss AG, Oberkochen, Germany) is used to pattern the nanostructures on a micromachined silicon mold. The mold is purchased with custom microstructures fabricated by conventional photolithography.

These structures consist of two facing U-shaped microchannels, separated by a 100 µm gap. The microchannels are 500 µm wide and 50 µm deep. Properly spaced pillars, with a diameter of 50 µm, prevent the collapse of the microchannels during the replica sealing process. In order to test different kinds of nanostructures, two different nanopatterns are milled, with a FIB, on the gap of two silicon molds. One is a nearly 2 µm long nanochannel connected to two trapezoidal access regions, and it results in being similar to an hourglass; the other is a funnel structure with a nanometric tip that is nearly 6 µm long. Both these nanometric structures have an approximately triangular section with a height of 80 nm and a width of nearly 800 nm. These structures constitute the only fluidic connection between the two U-shaped microchannels.

Figure 1B shows the second step: i.e., the procedure for creating the negative replica. During this step, we poured the degassed 10:1 mixture (prepolymer and curing agent *w/w* ratio) of standard PDMS (DOWSIL™ 184 Silicone Elastomer Kit, Midland, MI, USA) on the silicon master. After 4 h of curing at 60 °C in the oven, the cross-linked polymer is peeled-off from the mold. In order to favor this process, we deposited 1H,1H,2H,2H-perfluorooctyltrichlorosilane (FOTS—Sigma Aldrich, St. Louis, MI, USA) onto the mold by vapor phase. The negative replica, that we obtain, has the patterned structures in relief; thus, a second replica molding process is needed to obtain a positive polymeric copy of the silicon mold.

This third step is shown in Figure 1C. We used several formulations of the same polymer: standard (DOWSIL^TM^ 184) PDMS 1:1 and PDMS 10:1 and h-PDMS. The h-PDMS was prepared by mixing four compounds: (i) 1.7 g of a vinyl PDMS prepolymer (VDT-731, Gelest Corp. Morrisville, PA, USA), (ii) 4.5 µL of Pt-based catalyst (platinum divinyltetramethyldisiloxane, Gelest Corp.), (iii) 0.05 g of modulator (1,3,5,7-tetravinyl-1,3,5,7-tetramethyl cyclotetrasiloxane, Gelest Corp.) and (iv) 0.3 g of hydrosilane prepolymer (HMS-301 Gelest Corp.). Firstly, a drop of h-PDMS was slowly dispensed with a syringe and a 30 gauge needle on the region of the negative replica with the nanostructures. The volume was sized to confine the h-PDMS only in this region. We refer to this approach as “focused drop-casting”. Then, the deposited h-PDMS drop was cured for 30 min at 70 °C. A layer of PDMS 1:1 was poured all over the negative replica, spun at 1000 rpm for 60 s and cured for an hour. Finally, a layer of PDMS 10:1 was deposited and cured for 4 h at 70 °C.

The positive replica was peeled off the mold and further incubated at 150 °C overnight to ensure complete cross-linking [36].

The fluidic access for the working solutions was obtained by drilling holes with a needle in the reservoirs. In the final step, the replica was exposed to an oxygen plasma (Tucano TUC-1B-MF, Gambetti Kenologia s.r.l., Binasco (MI), Italy) at 50 W for 30 s and brought into contact with a glass coverslip, thus obtaining a sealed and water-tight nanofluidic device (Figure 1D) ready to be filled with sample solutions for sensing experiments.

### 2.2. Fabrication of Control Devices

The necessity and the effectiveness of this “focused drop-casting” method, which results in a patchwork surface, was tested by fabricating two control devices with procedures analogous to that described in Section 2.1, one without using h-PDMS but only PDMS 1:1 and 10:1, and another without confining the h-PDMS that thus completely replaced the PDMS 1:1 layer.

### 2.3. Optical and Atomic Force Microscopy (AFM) Characterization of the Device

Optical microscope images of the devices were acquired during and after the fabrication with a BX51 microscope (Olympus, Tokyo, Japan) equipped with an F-View II camera controlled by CellB Software. For obtaining information on the effect of the sealing process on the nanostructures, both the patchwork replica and the one without h-PDMS were brought into conformational contact with a clean glass substrate. 81 Norland Optical Adhesive (NOA, Cranbury, NJ, USA) resin, previously diluted in acetone (1:1 *v*/*v*), was inserted in one of the two microchannels, let fill the nanochannel, and cured under ultraviolet (UV) irradiation for 2 h with a 365 nm UV lamp (Biolink, Vilber Lourmat, Marne-la-Vallée, France). After curing, we peeled off the replicas and obtained two casts that were imaged by atomic force microscopy (AFM) that provided information on the geometry and size of the nanochannels after the device closure. The AFM measurements were performed in tapping mode by a Dimension 3100 microscope (Veeco Instruments, Plainview, NY, USA) and silicon nitride tip (Olympus). The software WSxM [37] was used to analyze and process the images.

### 2.4. Electrical Measurements

After filling the device with KCl 1 M, current–voltage (I–V) curves were acquired by using an electronic amplifier (EPC 10 Usb, Heka Electronik, Multi Channel Systems MCS GmbH, Reutlingen, Germany) and Ag/AgCl electrodes. All the electrical measurements were performed in a Faraday cage for minimizing the external noise. The sampling rate was 10 kHz, and traces were filtered by low-pass Bessel filter with cut-off at 2.9 kHz.

## 3. Results and Discussion

**Figure 2:**
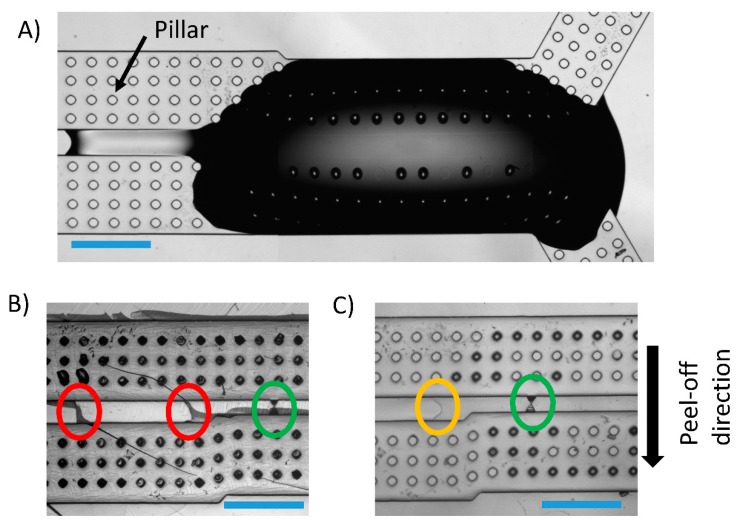
Comparison of replicas produced using hard poly(dimethylsiloxane) (h-PDMS) with the “focused-drop casting” approach and with h-PDMS all over the mold. The nanostructure patterned on the gap separating the two microchannels consists in a short nanochannel linking two trapezoidal access regions (green circle). (**A**) Optical microscope image of the polymeric negative replica with a drop of h-PDMS spread in correspondence of the nanopatterned region. (**B**) Picture of a positive polymeric replica fabricated depositing a layer of h-PDMS all over the negative replica surface. This method generates undesired deep cracks (red circles). (**C**) Picture of the positive replica obtained using h-PDMS dripped in a confined region. No cracks are visible. Here, it is possible to observe the interface between the h-PDMS patch and standard PDMS 1:1 (yellow circle). Scale bar 500 µm.

The optical investigations, performed during and after the fabrication procedure, confirmed that the “focused drop-casting” method was effective in confining the h-PDMS in the region of the nanochannel. Figure 2A shows the h-PDMS drop on the negative polymeric replica spreading along the gap between the microchannels, covering the nanostructure, but not invading the regions that extend in the direction orthogonal to the channels.

The h-PDMS remains confined because of the shape of the microchannels that act as a guide, hindering the polymer from spreading out of them. This material results confined in this region, even if other layers of PDMS (first 1:1 and then 10:1) are deposited over it, to produce the composite replica. We deposited a layer of PDMS 1:1, over the h-PDMS drop, in order to decrease progressively the stiffness of the material from h-PDMS to PDMS 10:1. In fact, it is demonstrated that increasing the curing agent to prepolymer base ratio and the curing time results in a stiffer material [38]. The planar heterogeneity of the material is visible (as a region with different contrast, see Figure 2C—yellow circle), observing the surface of the positive replica with a microscope after the curing process. Thus, the patchwork approach allows obtaining a replica stiffer in the nanostructured area, but more flexible in the other regions of the device. By choosing, as peel-off direction, the one that reduces the bending of the h-PDMS (shown by the black arrow in Figure 2C), we obtained an easy replica release without the formation of undesired cracks. On the contrary, as shown in Figure 2B, the device produced with h-PDMS, without using the “focused drop-casting” strategy, shows several deep cracks (red circles) because of its excessive stiffness. These cracks represent competitive points of connection between the two microchannels. They were not present on the negative replica and resulted in a discarded device, e.g. for electrical biosensing applications.

**Figure 3:**
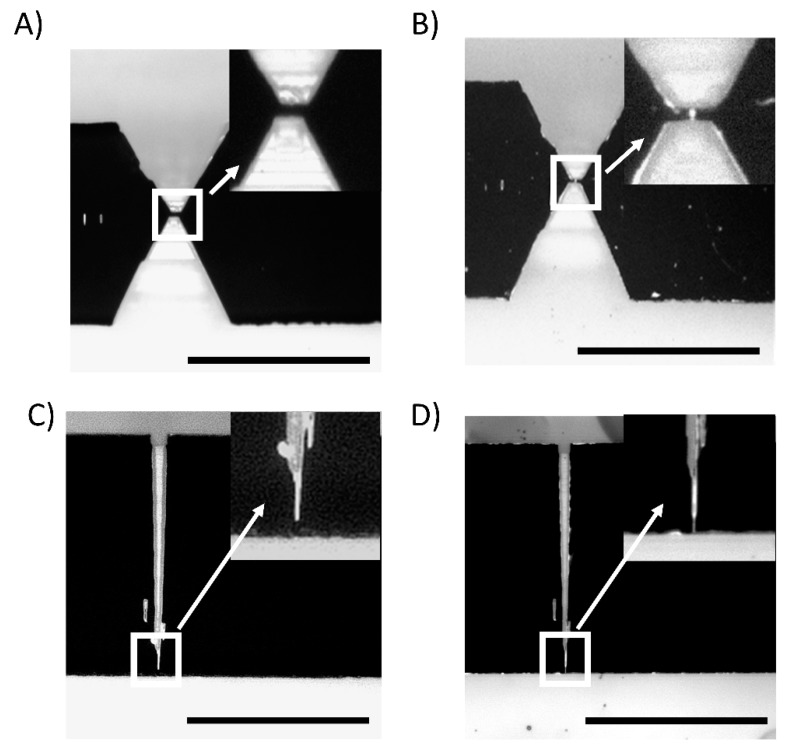
Nanochannels after the bonding procedure. Optical microscope images of (**A**,**C**) hourglass and funnel-shaped channels of a nanofluidic device made with standard PDMS and (**B**,**D**) made using h-PDMS and the focused drop-casting approach. Images’ insets show a zoomed view of the nanochannel region, allowing us to observe if they collapse or not. Scale bar 100 μm.

As to the necessity of using h-PDMS, we compared the devices created with the patchwork approach to the ones where we did not use h-PDMS. Figure 3 shows reflection optical microscopy images of the sealed devices with all the combinations of materials and geometries explored.

In all the cases, one can notice the microscale features that are contrasted by the difference of refractive index of the trapped air with respect to the polymer. However, the nanometric regions are visible only in devices made with h-PDMS and the “focused drop-casting” method (Figure 3B,D) while the lack of contrast for these features in the devices without h-PDMS, i.e., made only of PDMS 1:1 and PDMS 10:1, (Figure 3A,C) indicates no trapped air and thus the collapse of the nanochannels. We deduce that the stiffness of h-PDMS allows overcoming the attraction between the polymeric replica and the glass coverslip caused by surface interactions, thus preventing the main cause of the roof-collapse phenomenon [30,31,39,40].

### 3.1. AFM-Based Analysis of the Dimensions of the Casts

AFM was used to analyze the topography of NOA casts of the sealed funnel-shaped channels and also to determine the impact of using h-PDMS on the transposition of the mold geometry to the sealed device.

With this strategy, we estimated the dimensions of the nanochannel after closure as, due to “roof collapse,” the nanostructure sizes change significantly before and after sealing.

**Figure 4:**
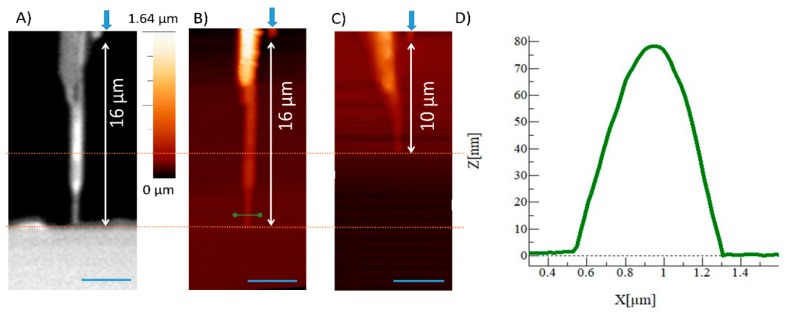
Atomic force microscopy (AFM) measurements. (**A**) Optical microscope image of the final part of nanochannel with the funnel geometry made with h-PDMS and the patchwork approach. AFM images of the NOA (Norland Optical Adhesive) cast of a funnel-shaped nanochannel made with (**B**) and without h-PDMS (**C**). The green line in (**B**) corresponds to the height profile, reported in (**D**), for the nanochannel made by using h-PDMS and the “focused-drop casting” strategy. Scale bar 4 μm.

Figure 4 shows the topography of casts obtained through the procedure described in Section 2.3 for devices fabricated by dripping h-PDMS with the patchwork approach and by using only standard PDMS (1:1 and 10:1), respectively.

The collapse occurred in the latter can be deduced by the measure of the channel length from a reference point (blue arrow in Figure 4B,C) to its tip. The measurements show that the replica made using standard PDMS has a cast 6 μm shorter, thus confirming the observations made by optical microscopy. From the topography of the cast near the funnel tip (Figure 4B—green line) it is possible to trace a profile (Figure 4D) from which we can estimate nanochannels’ dimensions. The NOA cast cross-section can be approximated with a triangle of height 80 nm and a base width of nearly 800 nm.

### 3.2. Electrical Characterization of Devices

**Figure 5:**
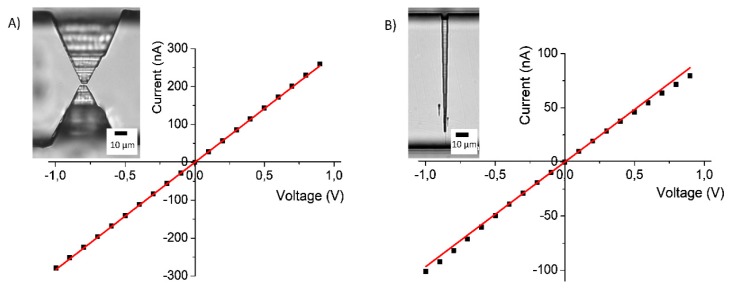
Electrical device characterization: (**A**) current-voltage (I-V) curve of the nanofluidic device with an hourglass-shaped structure and a short single nanochannel. (**B**) I–V curve of the nanofluidic device with a funnel-shaped nanochannel. The red lines represent linear fitting cur.

Finally, in order to test the devices’ functionality, we characterized them electrically by measuring I–V curves both for the funnel and for the hourglass geometry fabricated using the patchwork approach. Figure 5A,B show the curves acquired in the range ±1 V. In the case of the hourglass geometry, the curve is symmetric.

We estimated the nanochannel resistance by a linear fit of the experimental points (red line), obtaining (3.5 ± 0.1) MΩ and, as expected, the curve had an ohmic behavior while the I–V curve of the funnel geometry showed a slightly rectifying behavior probably due to the asymmetry of the structure [41]. In this case, the figure estimated for the resistance was (10.3 ± 0.1) MΩ. Then, we used the resistance values measured experimentally to verify the correspondence of the nanochannel sizes measured on the NOA casts with those of a sealed PDMS device. Considering a resistivity value of about 0.07 Ωm for a 1 M KCl solution, we inferred the dimensions of the sealed nanostructures for both device geometries: hourglass and funnel. We approximated the nanochannels’ cross-section with a triangle, a reasonable assumption considering the profile reported in Figure 4D. The measured resistance values were consistent with the cross-section dimensions estimated for the profile of the NOA cast, i.e., a base around 800 nm, a height of 80 nm, and a length of 2 µm for the short nanochannel of the hourglass geometry and 6 µm for the funnel. Thus, both the resistance values demonstrated the good agreement between the nanostructures fabricated on the mold and on the positive replica, confirming the fidelity of the “focused-drop-casting” approach in replicating nanostructures, and proved the efficiency of this method for producing devices not affected by leakage problems, a crucial advantage when used for electrokinetic measurements.

The polymeric sub-100 nm nanostructures fabricated with this approach could be exploited for a variety of applications and, in particular, for the development of a new class of nanosensors based on nanofluifìdic structures, such as those used for the detection of single nano-sized objects [42].

## 4. Conclusions

We have presented a novel approach for the fabrication, by soft lithography, of polymeric nanofluidic devices with nanochannels of different geometries. Sub-100 nm structures, with an aspect ratio of less than 0.1, have been obtained in crack-free devices without incurring in “roof collapse” phenomena. The proposed approach modulates the stiffness of the polymer across the device surface in order to increase its value only in the regions with a geometry prone to collapse during the sealing process. By maintaining overall flexibility, we can avoid the formation of cracks when the polymeric replica is peeled from its master. The geometry of the devices and their electrical properties have been characterized, showing that our approach can be used to obtain the characteristics required in the fabrication of nanofluidic sensors. In conclusion, our method provides a simple and effective solution for producing polymeric nanofluidic systems, reducing their cost, and improving their reliability.

## References

[1] Zhang M., Yang J., Cai Z., Feng Y., Wang Y., Zhang D., Pan X. (2019). Detection of engineered nanoparticles in aquatic environments: Current status and challenges in enrichment, separation, and analysis. Environ. Sci. Nano.

[2] Nie X., Liu H., Pan Z., Ahmed S., Shen Q., Yang J., Pan J., Pang J., Li C., Xia X. (2019). Recognition of plastic nanoparticles using a single gold nanopore fabricated at the tip of a glass nanopipette. Chem. Commun..

[3] Wei R., Gatterdam V., Wieneke R., Tampe R., Rant U. (2012). Stochastic sensing of proteins with receptor-modified solid-state nanopores. Nat. Nanotechnol..

[4] Han A., Creus M., Schurmann G., Linder V., Ward T., de Rooij N., Staufer U. (2008). Label-free detection of single protein molecules and protein-protein interactions using synthetic nanopores. Anal. Chem..

[5] Fanzio P., Mussi V., Menotta M., Firpo G., Repetto L., Guida P., Angeli E., Magnani M., Valbusa U. (2015). Selective protein detection with a dsLNA-functionalized nanopore. Biosens. Bioelectron..

[6] Harms Z., Mogensen K., Nunes P., Zhou K., Hildenbrand B., Mitra I., Tan Z., Zlotnick A., Kutter J., Jacobson S. (2011). Nanofluidic Devices with Two Pores in Series for Resistive-Pulse Sensing of Single Virus Capsids. Anal. Chem..

[7] Mitra A., Deutsch B., Ignatovich F., Dykes C., Novotny L. (2010). Nano-optofluidic Detection of Single Viruses and Nanoparticles. Acs Nano.

[8] Yang L., Yamamoto T. (2016). Quantification of Virus Particles Using Nanopore-Based Resistive-Pulse Sensing Techniques. Front. Microbiol..

[9] Aizel K., Agache V., Pudda C., Bottausci F., Fraisseix C., Bruniaux J., Navarro F., Fouillet Y. (2013). Enrichment of nanoparticles and bacteria using electroless and manual actuation modes of a bypass nanofluidic device. Lab Chip.

[10] Wang Z., Han T., Jeon T., Park S., Kim S. (2013). Rapid detection and quantification of bacteria using an integrated micro/nanofluidic device. Sens. Actuators B Chem..

[11] Fan R., Karnik R., Yue M., Li D., Majumdar A., Yang P. (2005). DNA translocation in inorganic nanotubes. Nano Lett..

[12] Mussi V., Fanzio P., Repetto L., Firpo G., Scaruffi P., Stigliani S., Tonini G., Valbusa U. (2010). DNA-functionalized solid state nanopore for biosensing. Nanotechnology.

[13] Menard L., Ramsey J. (2013). Electrokinetically-Driven Transport of DNA through Focused Ion Beam Milled Nanofluidic Channels. Anal. Chem..

[14] Piruska A., Gong M., Sweedler J., Bohn P. (2010). Nanofluidics in chemical analysis. Chem. Soc. Rev..

[15] Majd S., Yusko E., Billeh Y., Macrae M., Yang J., Mayer M. (2010). Applications of biological pores in nanomedicine, sensing, and nanoelectronics. Curr. Opin. Biotechnol..

[16] Venkatesan B., Bashir R. (2011). Nanopore sensors for nucleic acid analysis. Nat. Nanotechnol..

[17] Kim M., McNally B., Murata K., Meller A. (2007). Characteristics of solid-state nanometre pores fabricated using a transmission electron microscope. Nanotechnology.

[18] Hinkle P., Westerhof T., Qiu Y., Mallin D., Wallace M., Nelson E., Taborek P., Siwy Z. (2017). A hybrid resistive pulse-optical detection platform for microfluidic experiments. Sci. Rep..

[19] Pedone D., Langecker M., Munzer A., Wei R., Nagel R., Rant U. (2010). Fabrication and electrical characterization of a pore-cavity-pore device. J. Phys. Condens. Matter.

[20] Gates B., Xu Q., Stewart M., Ryan D., Willson C., Whitesides G. (2005). New approaches to nanofabrication: Molding, printing, and other techniques. Chem. Rev..

[21] Lo C., Aref T., Bezryadin A. (2006). Fabrication of symmetric sub-5 nm nanopores using focused ion and electron beams. Nanotechnology.

[22] Tseng A. (2004). Recent developments in micromilling using focused ion beam technology. J. Micromec. Microeng..

[23] Altissimo M. (2010). E-beam lithography for micro-/nanofabrication. Biomicrofluidics.

[24] Liao Y., Cheng Y., Liu C., Song J., He F., Shen Y., Chen D., Xu Z., Fan Z., Wei X. (2013). Direct laser writing of sub-50 nm nanofluidic channels buried in glass for three-dimensional micro-nanofluidic integration. Lab Chip.

[25] Hui A.P., Qin S.J., Li W.J., Wang M.Y. High aspect ratio nano fluidic channels by laser-controlled fracturing. Proceedings of the Fifteenth IEEE International Conference on Micro Electro Mechanical Systems.

[26] Chantiwas R., Hupert M., Pullagurla S., Balamurugan S., Tamarit-Lopez J., Park S., Datta P., Goettert J., Cho Y., Soper S. (2010). Simple replication methods for producing nanoslits in thermoplastics and the transport dynamics of double-stranded DNA through these slits. Lab Chip.

[27] McDonald J., Duffy D., Anderson J., Chiu D., Wu H., Schueller O., Whitesides G. (2000). Fabrication of microfluidic systems in poly(dimethylsiloxane). Electrophoresis.

[28] Manneschi C., Fanzio P., Ala-Nissila T., Angeli E., Repetto L., Firpo G., Valbusa U. (2014). Stretching of DNA confined in nanochannels with charged walls. Biomicrofluidics.

[29] Odom T., Love J., Wolfe D., Paul K., Whitesides G. (2002). Improved pattern transfer in soft lithography using composite stamps. Langmuir.

[30] Zhou W., Huang Y., Menard E., Aluru N., Rogers J., Alleyne A. (2005). Mechanism for stamp collapse in soft lithography. Appl. Phys. Lett..

[31] Huang Y., Zhou W., Hsia K., Menard E., Park J., Rogers J., Alleyne A. (2005). Stamp collapse in soft lithography. Langmuir.

[32] Schmid H., Michel B. (2000). Siloxane polymers for high-resolution, high-accuracy soft lithography. Macromolecules.

[33] Choi K., Rogers J. (2003). A photocurable poly(dimethylsiloxane) chemistry designed for soft lithographic molding and printing in the nanometer regime. J. Am. Chem. Soc..

[34] Huelsen C., Probst J., Loechel B. (2014). Replication of sub-100 nm structures using h- and s-PDMS composite stamps. Microsyst. Technol..

[35] Kim K., Song N., Choo B., Pribat D., Jang J., Park K., Yoo S. (2008). Mechanical characteristics of the hard-polydimethylsiloxane for smart lithography. Ekc2008 Proc. EU-Korea Conf. Sci. Technol..

[36] Huh D., Mills K., Zhu X., Burns M., Thouless M., Takayama S. (2007). Tuneable elastomeric nanochannels for nanofluidic manipulation. Nat. Mater..

[37] Horcas I., Fernandez R., Gomez-Rodriguez J.M., Colchero J., Gomez-Herrero J., Baro A.M. (2007). WSXM: A software for scanning probe microscopy and a tool for nanotechnology. Rev. Sci. Instrum..

[38] Seghir R., Arscott S. (2015). Extended PDMS stiffness range for flexible systems. Sens. Actuators A Phys..

[39] Mao P., Han J. (2005). Fabrication and characterization of 20 nm planar nanofluidic channels by glass-glass and glass-silicon bonding. Lab Chip.

[40] Pinti M., Prakash S. Fabrication of Hybrid Micro-Nanofluidic Devices with Centimeter Long Ultra-Low Aspect Ratio Nanochannels. Proceedings of the Asme International Mechanical Engineering Congress and Exposition.

[41] Hou X., Dong H., Zhu D., Jiang L. (2010). Fabrication of Stable Single Nanochannels with Controllable Ionic Rectification. Small.

[42] Angeli E., Volpe A., Fanzio P., Repetto L., Firpo G., Guida P., Lo Savio R., Wanunu M., Valbusa U. (2015). Simultaneous Electro-Optical Tracking for Nanoparticle Recognition and Counting. Nano Lett..

